# Sex-Related Pain Behavioral Differences following Unilateral NGF Injections in a Rat Model of Low Back Pain

**DOI:** 10.3390/biology11060924

**Published:** 2022-06-16

**Authors:** Michael Syrett, Nicholas R. Reed, William R. Reed, Madison L. Richey, Andrey Frolov, Joshua W. Little

**Affiliations:** 1 Saint Louis University School of Medicine, 1402 South Grand Blvd., Saint Louis, MO 63104, USA; sa177404@atsu.edu (M.S.); nickreed44@gmail.com (N.R.R.); mlrq67@umsystem.edu (M.L.R.); andrey.frolov@health.slu.edu (A.F.); 2 Department of Physical Therapy, University of Alabama at Birmingham, 1720 2nd Ave. South, Birmingham, AL 35294, USA; wreed@uab.edu

**Keywords:** sex differences, females, nerve growth factor, low back pain, musculoskeletal pain, mechanical hypersensitivity, mechanical hyperalgesia, pain

## Abstract

**Simple Summary:**

Low back pain (LBP) is a worldwide problem that requires additional studies to better identify its underlying causes. One obstacle encountered is that female-experienced LBP is likely biologically different from male-experienced LBP. Studying the potential sex-differences in animal models may provide a better understanding of the causes of LBP and help to identify new treatments for LBP. We used an animal model to investigate the differences in male and female rats when back muscles on one side were injected with a painful substance called nerve growth factor (NGF). These injections led to sex differences in the behavioral responses to painful stimuli (hyperalgesia) and non-painful stimuli (mechanical hypersensitivity). Ipsilateral low back deep hyperalgesia and superficial mechanical hypersensitivity lasted longer in females compared with males. Moreover, female rats had bilateral deep mechanical hyperalgesia and cutaneous trunk mechanical hypersensitivity, unlike males. Our results are the first to demonstrate that there are sex differences in an animal model of LBP induced by the painful substance NGF. Future studies may help to reveal the underlying biological mechanisms of LBP to create better treatments to attempt to help those suffering with LBP.

**Abstract:**

Low back pain (LBP) is a globally prevalent and costly societal problem with multifactorial etiologies and incompletely understood pathophysiological mechanisms. To address such shortcomings regarding the role of neurotrophins in the underlying mechanisms of pain, an LBP model was developed in rats involving two unilateral intramuscular injections of nerve growth factor (NGF) into deep trunk muscles. To date, behavioral investigations of this NGF-LBP model have been limited, especially as it pertains to female pain behaviors. This study compared mechanical sensitivity to noxious (hyperalgesia) and non-noxious (hypersensitivity) stimuli in control and NGF-injected male and female rats through pain resolution. Although the baseline testing revealed no differences between males and females, NGF-injected females demonstrated prolonged ipsilateral deep trunk mechanical hyperalgesia that resolved seven days later than males. Moreover, females showed bilateral trunk mechanical sensitivity to noxious and non-noxious stimuli compared to only ipsilateral behaviors in males. Sex differences were also observed in the severity of behavioral responses, with females displaying greater mean differences from baseline at several timepoints. Overall, these NGF-LBP behavioral findings mirror some of the sex differences reported in the clinical presentation of LBP and accentuate the translatability of this NGF-LBP model. Future studies using this LBP-NGF model could help to elucidate the neurobiological mechanisms responsible for the development, severity, and/or resolution of muscular LBP as well as to provide insights into the processes governing the transition from acute to chronic LBP.

## 1. Introduction

Low back pain (LBP) is clinically complex, poorly managed, and is the leading cause for global disability [1]. Although LBP is often considered to be a structural disorder affecting the vertebrae and/or joints of the lumbar spinal column, the musculature of the deep back frequently contributes to the pain in this region [2,3,4,5]. Musculoskeletal pain presents differently between the sexes clinically, which only further complicates the ability to determine their respective neurobiological mechanism(s) [6,7,8]. In particular, human female subjects are reported as being more sensitive to musculoskeletal pain and having a greater risk of developing widespread central pain syndromes [9,10,11,12,13,14]. Although LBP is a substantially unresolved medical need, relatively little attention is devoted to understanding its underlying pathophysiological mechanisms [15]. Neurobiological pain mechanisms are difficult to investigate in humans and are often better elucidated and more easily characterized using preclinical animal models. However, current preclinical LBP models are relatively limited, with most of them examining discogenic pathologies [16,17], despite muscle being identified as a common source of LBP [3,16,17,18]. Importantly, regardless of the etiology of LBP, there appears to be an increasing clinical interest in the role that nerve growth factor (NGF) plays, as anti-NGF approaches in recent LBP clinical trials demonstrated efficacy in the management of LBP [19,20,21]. Therefore, preclinical models that mimic the clinical features of LBP become of great importance for the development of novel pharmacological and nonpharmacological strategies to effectively treat LBP.

The best-characterized lumbar musculature are the erector spinae and multifidi muscles, as they have the architecture (e.g., short muscle fiber length and large cross-sectional area) and physiological properties (e.g., specialized sarcomere length operating range) that suggest key roles in lumbar spinal stabilization and in the development of LBP [22]. The physiological roles of the erector spinae and multifidi muscles in the lumbar spine may be best modeled in rats. Rat epaxial muscles include the erector spinae, multifidus, and the transversospinalis muscle groups, with the multifidi muscles most prominent in the lower lumbar spine [23]. Studies of the structure (e.g., sarcomere length) and function (e.g., passive elastic moduli) of rat multifidi and erector spinae indicate that rat trunk muscles are more similar to humans than either mice or rabbits [23,24]. Additionally, secondary pathological changes in trunk musculature that occur after spinal degeneration in humans are also reported in rats [25]. As the structural and intrinsic properties of muscles are the best predictors of muscle function [26], additional characterization of trunk muscle pain in rats could become essential in revealing key underlying pathophysiological mechanisms of LBP in humans.

NGF is a neurotrophin involved in neuronal growth and survival during development while remaining important in pain transduction throughout adulthood [27]. Recently, an LBP model was developed that involves two unilateral NGF injections (separated by 5 days) into the multifidus muscle of adult male rats [28]. Following the second unilateral NGF trunk injection, persistent ipsilateral trunk mechanical hyperalgesia (LBP) is detected through at least a 14-day time course [28]. Unfortunately, in these earlier unilateral NGF-LBP studies [28,29,30], the time to the resolution of NGF-related pain behaviors was not determined and female rats were not included, thereby preventing the characterization of potential sex differences (which are translationally relevant to clinical LBP). Thus, we recently expanded this unilateral NGF-LBP model by demonstrating that other somatosensory alterations, including delayed cutaneous mechanical hypersensitivity to touch, develop during this time course in male rats [31]. We conducted the first investigation using female rats in this NGF-LBP model [32], but the focus of this particular study was on the anti-hyperalgesic effects of simulated spinal manual therapy, and it contained no direct comparisons of female and male mechanical sensitivity. However, in healthy human participants, NGF-induced mechanical sensitization and pain during oral function was found to be significantly greater in women compared to men in healthy human participants [8]. While the mechanisms underlying these NGF-induced sex-related differences are poorly understood, early evidence suggests increases in peripheral NMDA-receptor expression without an increase in nerve fiber density [8,33,34]. Therefore, to better address this gap in the literature regarding the potential sex-related behavioral differences in the unilateral NGF-LBP rat model, we performed this initial comparative study aiming to uncover putative male and female differences in somatosensory alterations after two unilateral NGF injections into the deep trunk musculature. We examined time-dependent differences in the baseline, onset, severity (i.e., the magnitude of change from baseline), laterality (i.e., ipsilateral and contralateral), and resolution of pain behavioral responses. The somatosensory behavioral assays performed in the NGF-LBP model were based on our previous study [31] and are similar to some of the standardized quantitative somatosensory tests typically performed in the low back and remote anatomical regions in clinical studies of LBP patients [35,36]. The mechanical sensitivity assays included remote (i.e., hindpaw) and local (i.e., trunk) cutaneous stimulation and local (i.e., trunk) deep muscle mechanical stimulation via algometer testing [31]. A detailed characterization of altered LBP somatosensory behaviors in females with direct comparisons to males could provide important evidence for the potential of sex differences in LBP as well as guidance to investigations of the neurobiological mechanisms responsible for these phenomena. We anticipated that following unilateral intra-muscular NGF injections females would demonstrate greater mechanical sensitivity, bilateral hypersensitivity, and prolonged LBP behaviors compared to males.

## 2. Methods

All experiments conformed to the guidelines for animal research of the National Institutes of Health and the ethical guidelines for pain research on animals of the International Association for the Study of Pain. All described experimental procedures were reviewed and approved by the Institutional Animal Care and Use Committee of Saint Louis University. Female (*n* = 16) and male (*n* = 16) Sprague Dawley rats (Envigo, Indianapolis, IN; 215–225 g at study onset, 8 weeks at the start of experiments) were used in these studies. In addition, we designed our female and male NGF-LBP experiments to be performed using the same protocols on the same day and with the same lab personnel for meaningful direct comparisons. This experimental strategy involved a thorough cleaning of all lab equipment between the testing of different sexes. Rats were housed in the same room with *n* = 3 per cage with males and females in separate cages and food and water provided ad libitum. Animal rooms were in climate-controlled conditions with 12-h light–dark cycles.

### 2.1. NGF-LBP Model

The lumbar region was shaved 1 day before baseline testing. Two NGF injections were administered 5 days apart (D0 and D5) into the left trunk (multifidus) muscles (3 mm from the L5 spinous process, with the injection site being marked with indelible marker), and all injections were performed under isoflurane anesthesia [28,32]. Human recombinant NGF (Sigma-Aldrich catalog # N1408) was dissolved in 50 μL of phosphate-buffered saline (PBS) at 0.8 μM and a pH of 7.2–7.3. Control rats received 50 μL of vehicle (PBS). This NGF concentration in the low back muscle has been shown to cause mechanical sensitivity in rats and humans [28,32]. Rats were acclimated to the testing environment and laboratory examiners for 2–3 days prior to testing. All rats were randomly assigned into groups, and the examiners were blinded to the animal groups. Behavior was measured at baseline, D0, (pre-1st injection); post-1st injection at selected time points, including D2 and D5 (pre-2nd injection); and post-2nd injection at D5 + 4 h, D7, D10, D14, D17, D21, D24, and D28. The established NGF model requires two injections to induce trunk pain [28,29,30,31,32]. The first injection induces an acute trunk pain response (hyperalgesia) that typically peaks by 2 days (D2) and returns to baseline by D5. This acts as a priming effect so that after the second injection there is a prolonged hyperalgesia that is significant by 4 h and continues at least through D14 [28,29,30]. Behavioral assays were always performed from the least invasive to most invasive and from superficial to deep (i.e., cutaneous sensitivity testing prior to deep trunk algometer testing) in a similar fashion to quantitative sensory testing in humans [31,37].

### 2.2. Cutaneous Hindpaw Mechanical Behavioral Assay

After 20 min of acclimation on a mesh platform in clear Plexiglas animal enclosures (17 cm depth × 69 cm length × 14 cm height; IITC Life Science, Woodland Hills, CA, USA), remote (hindpaw) cutaneous mechanical sensitivity was tested bilaterally at the midplantar surface using calibrated von Frey (VF) filaments (Stoelting, range: 3.61 [0.407 g] to 5.46 [26 g] bending force) and the up-and-down method [31,32,38]. This testing method requires three behavioral changes to occur from 1. negative response to positive response, 2. positive response to negative response, and 3. negative response to positive response. The series of positive and negative responses to the applied filaments were then entered into a formula to determine the 50% withdrawal threshold, or mean PWT. The 50% PWT was determined as 10[Xf + k(∆)]/10,000, where Xf = last filament employed, k = Dixon value, and ∆ = stimulus interval. A positive response was a sharp/brisk withdrawal of the hindpaw while the mechanical stimulation (i.e., calibrated filament) was against the paw. The data were log transformed (log (g)) for analysis and also reported as mean PWT (g) [39]. The mean paw withdrawal threshold (PWT; g) and mean difference from baseline were reported for each group.

### 2.3. Cutaneous Trunk Mechanical Behavioral Assay

After completing the hindpaw testing and while the rats were still in the enclosure, the cutaneous trunk sensitivity was tested using calibrated VF filaments (1 g and 4 g) as previously reported [31,40]. The first applied trials were the ones with 1 g filaments followed by trials with 4 g filaments to the trunk skin at the L5 level bilaterally. Each filament was applied until bending occurred, held for 3 s, then repeated for 10 total trials (3–5 s break between stimuli). A positive response could include a skin twitch, aversive movements, or a postural position change. The number of positive responses was recorded and reported as the mean number of positive responses and mean difference from baseline for each group.

### 2.4. Algometer Deep Trunk Behavioral Assay

Deep trunk mechanical hyperalgesia was assessed using a small animal algometer (Smalgo^®^, Bioseb Instruments, Pinellas Park, FL) with a blunt 5 mm diameter tip probe applied bilaterally (2–3 trials/side; 2–3 min between trials) [31,32,41]. The blunt probe preferentially stimulates deeper muscle nociceptors [28]. Increasing mechanical probe pressure (g) was steadily applied until a pain-related behavior (i.e., withdrawal behavior, escape movements, and/or vocalization) was elicited or until an 800 g cutoff [28,31,32]. The threshold of response was recorded and reported as the mean withdrawal threshold (g) and mean difference from baseline for each group.

## 3. Statistical Analyses

Prior to beginning the experiments, all behavioral assays demonstrated consistency across multiple testing days in preliminary studies. Mechanical hypersensitivity was defined as significant (*p* < 0.05) reductions in mean PWT (g) or increases in mean positive responses to 1 g and 4 g VF filaments. Mechanical hyperalgesia was defined as a significant (*p* < 0.05) reduction in the mean withdrawal threshold with algometer testing (g).

A multivariate analysis was used to determine if there were any main or interaction effects of sex, side, examiners, and/or the order of testing sexes on any of the primary outcome measures. We also compared within and between sexes. We used a two-tailed two-way ANOVA followed by Bonferroni’s tests for the behavioral studies to make multiple comparisons at multiple time points compared to baseline within the same group. Tukey’s multiple comparisons were used to compare between groups at multiple time points. The severity (magnitude of change from baseline) and resolution of LBP behaviors were compared between males and females. The analyses used SPSS (v24) and GraphPad Prism (v6.04) reporting means ± SD. The sample sizes for the behavioral studies were based upon previous studies [28,32] to adequately power (0.80, α = 0.05, moderate effect size) the parametric statistical analyses. Statistical significance was defined as *p* < 0.05.

## 4. Results

### 4.1. Cutaneous Hindpaw Mechanical Hypersensitivity

**Figure 1:**
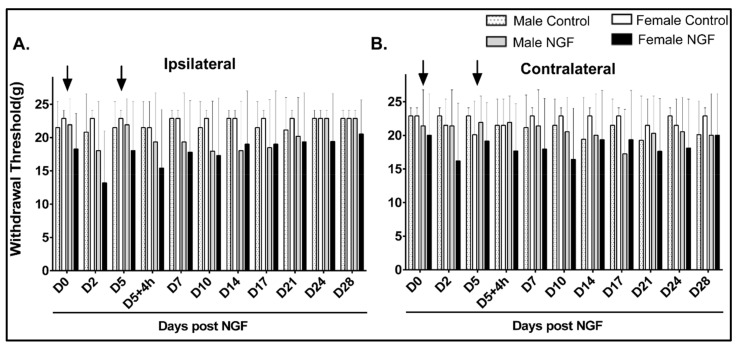
Behavioral responses to ipsilateral and contralateral cutaneous hindpaw mechanical stimuli were not altered during persistent low back pain in males and females. (**A**,**B**) Injections (arrows) of control (PBS) or NGF (0.8 μM) were not associated with ipsilateral (**A**) or contralateral (**B**) hindpaw mechanical hypersensitivity. There were no significant alterations in withdrawal thresholds to cutaneous stimuli from von Frey filaments in male or female rats compared to baseline (D0). No significant differences were detected at D0 when comparing all groups. Data are reported as means ± SD and analyzed by two-way ANOVA with Bonferroni’s comparisons to baseline within a group or two-way ANOVA with Tukey’s multiple comparisons between groups for *n* = 8/group.

An examination of cutaneous mechanical stimuli to the ipsilateral and contralateral hindpaws revealed that male and female rats in the control or NGF groups had no significant differences in the mechanical withdrawal thresholds at either hindpaw compared to D0 at baseline or any other time point assessed (Figure 1A,B; *p* > 0.05). An additional between-group analysis of severity (i.e., the mean difference from baseline) in the ipsilateral and contralateral hindpaw withdrawal threshold also demonstrated no significant differences between groups (data not shown).

### 4.2. Cutaneous Trunk Mechanical Hypersensitivity (1 g)

**Figure 2:**
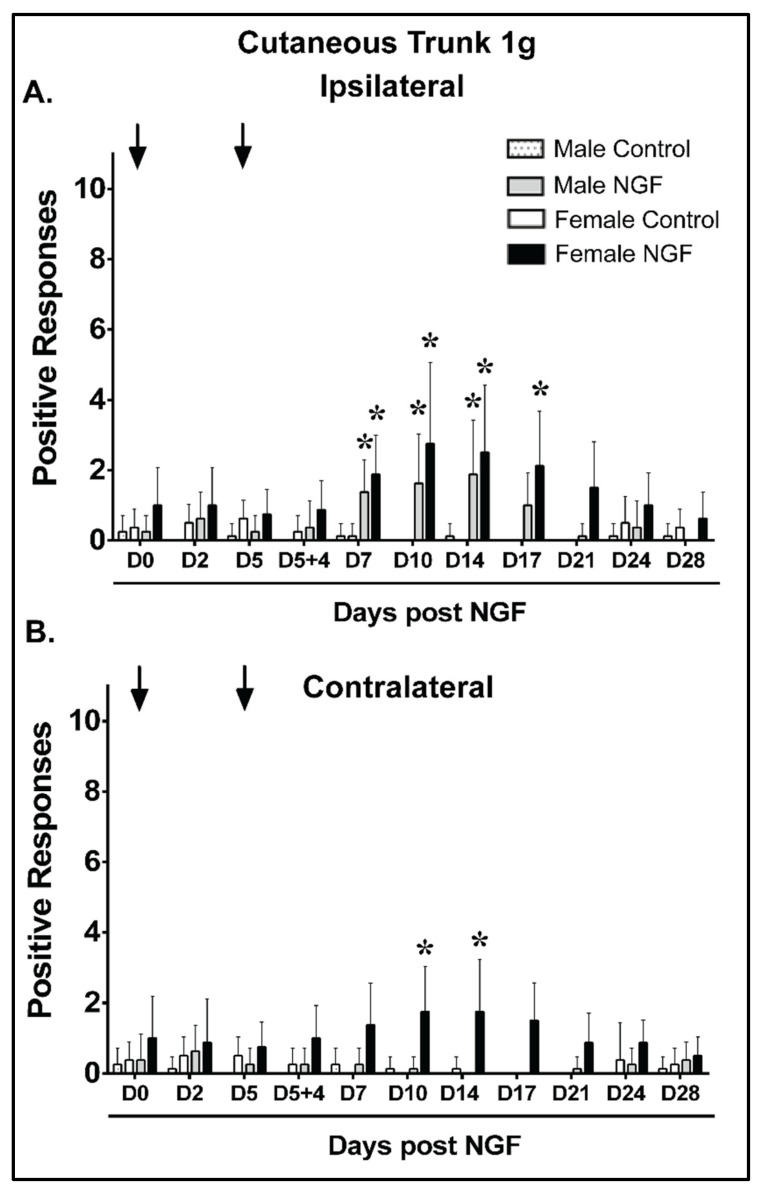
Females demonstrated prolonged and bilateral mechanical hypersensitivity to a 1 g cutaneous low back stimulus during persistent low back pain compared with males. (**A**) When compared to control (PBS), NGF injections (arrows, 0.8 μM) resulted in ipsilateral low back mechanical hypersensitivity with increased mean positive responses to a cutaneous stimulus for 1 g filament from D7 to D14 in male rats and from D7 to D17 in female rats compared to baseline (D0). (**B**) NGF injections were also associated with contralateral low back mechanical hypersensitivity with increased mean positive responses to a cutaneous 1 g filament stimulus at D10 and D14 in female rats only when compared to baseline (D0). No significant changes were noted in males compared to D0. No significant differences were detected at D0 when comparing all groups (**A**,**B**). Data are reported as means ± SD and analyzed by two-way ANOVA with Bonferroni’s comparisons to baseline within a group or two-way ANOVA with Tukey’s multiple comparisons between groups for *n* = 8/group. * *p* < 0.01 vs. D0 prior to injection.

The mean baseline responses to a 1 g cutaneous trunk mechanical stimulus applied ipsilaterally were not significantly different between males and females for any group (Figure 2A; *p* > 0.05). Male and female control rats had no significant positive response differences to 1 g ipsilateral trunk stimulation compared to D0 at any assessed time point (Figure 2A; *p* > 0.05). In contrast, male and female rats with NGF injections demonstrated significant ipsilateral mechanical hypersensitivity at D7, D10, and D14 in males (*p* < 0.01) and D7, D10, D14, and D17 in females compared to D0 (Figure 2A; *p* < 0.01). A between-group analysis of severity demonstrated that NGF males had greater ipsilateral increases than control males at D7, D10, and D14 and NGF females had greater ipsilateral responses than control females at D7, D10, D14, and D17 (Figure S1A). No significant differences in the mean difference from baseline were noted at any time points when compared between NGF males and females (Figure S1A). The ipsilateral cutaneous mechanical hypersensitivity resolved by D21 in NGF females compared to that of NGF males, which resolved by D17 (Figure 2A).

The mean baseline responses to a contralateral 1 g cutaneous trunk mechanical stimulus were not significantly different between males and females for any group (Figure 2B). Male and female control rats and male NGF rats had no significant contralateral differences compared to D0 at any time point assessed (Figure 2B; *p* > 0.05). In contrast, female rats that received NGF injections demonstrated a significant contralateral increase in mechanical sensitivity at D10 and D14 compared to D0 (Figure 2B, *p* < 0.01). A between-group analysis demonstrated that NGF females had greater responses than vehicle females at D10 and D14 (Supplementary Figure S1B). Significant differences (*p* < 0.01) in the mean change from baseline were detected between NGF males and females at D10 and D14 (Supplementary Figure S1B). The contralateral cutaneous mechanical hypersensitivity resolved by D17 in NGF females, which differed in relation to NGF males that demonstrated no contralateral mechanical hypersensitivity (Figure 2B).

### 4.3. Cutaneous Trunk Mechanical Hypersensitivity (4 g)

**Figure 3:**
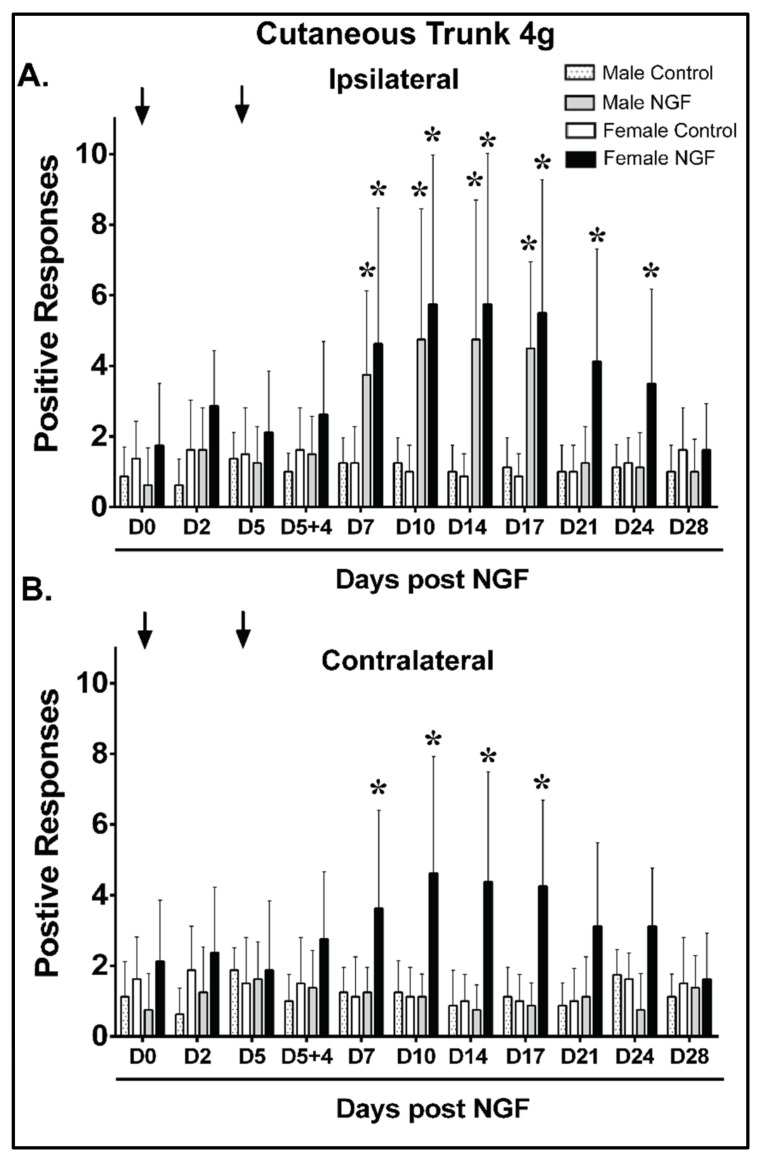
Females demonstrated prolonged and bilateral mechanical hypersensitivity to a 4 g cutaneous low back stimulus during persistent low back pain compared with males. (**A**) When compared to control (PBS), NGF injections (arrows, 0.8 μM) resulted in ipsilateral low back mechanical hypersensitivity with increased mean positive responses to a cutaneous 4 g filament stimulus from D7 to D17 in male rats and from D7 to D24 in female rats compared to baseline (D0). (**B**) NGF injections were also associated with contralateral low back mechanical hypersensitivity with increased mean positive response to a cutaneous 4 g filament stimulus at D7, D10, D14, and D17 in female rats compared to baseline (D0). No significant changes were noted in males compared to baseline at any time point. No significant differences were detected at D0 when comparing all groups (**A**,**B**). Data are reported as means ± SD and analyzed by two-way ANOVA with Bonferroni’s comparisons to baseline within a group or two-way ANOVA with Tukey’s multiple comparisons between groups for *n* = 8/group. * *p* < 0.01 vs. D0 prior to injection.

The mean ipsilateral baseline responses were not significantly different between males and females for any group with a 4 g cutaneous trunk mechanical stimulus (Figure 3A). Male and female control rats had no significant response ipsilaterally to a 4 g trunk mechanical stimulus compared to D0 at any time point (Figure 3A; *p* > 0.05). In contrast, male and female NGF rats demonstrated significant ipsilateral increases at D7, D10, D14, and D17 in males (*p* < 0.01; *n* = 8 rats/group) and D7, D10, D14, D17, D21, and D24 in females (Figure 3A; *p* < 0.01). A between-group analysis of severity demonstrated that NGF males had greater ipsilateral changes from baseline than control males at D7, D10, D14, and D17 and NGF females had greater ipsilateral responses than control females at D7, D10, D14, D17, D21, and D24 (Figure S2A). No significant differences in the magnitude of change were noted at any time points between NGF males and females (Figure S2A). The ipsilateral cutaneous hypersensitivity resolved by D28 in NGF females compared to NGF males that resolved by D21 (Figure 3A).

Baseline contralateral 4 g cutaneous trunk mechanical stimuli revealed no significant differences between males and females for any group (Figure 3B). Male and female control rats and NGF male rats had no significant differences compared to D0 at any time point assessed (Figure 3B; *p* > 0.05). In contrast, NGF female rats demonstrated significant mechanical sensitivity increases at D7, D10, D14, and D17 (Figure 3B; *p* < 0.01). An analysis of the mean differences from baseline demonstrated that NGF females had greater contralateral responses than control females at D7, D10, D14, and D17 (Figure S2B). Significant differences (*p* < 0.01) in the mean change from baseline were detected between NGF males and NGF females at D10, D14, and D17 (Figure S2B). The contralateral cutaneous mechanical hypersensitivity resolved by D21 in NGF females compared to NGF males, which demonstrated no contralateral hypersensitivity (Figure 3B).

### 4.4. Deep Trunk Mechanical Hyperalgesia

**Figure 4:**
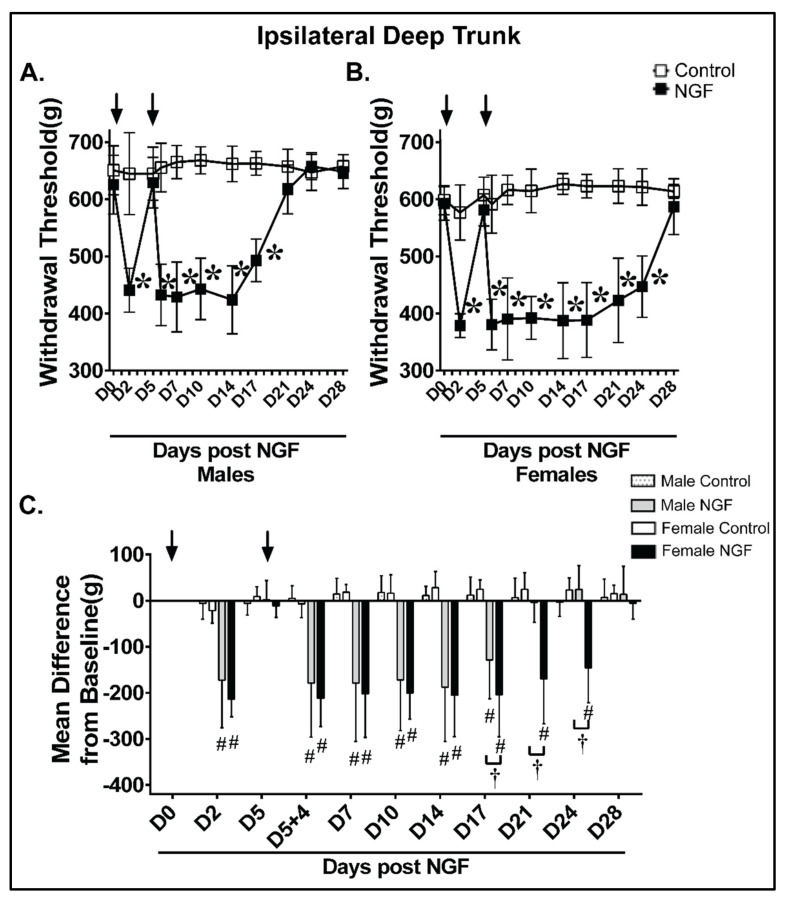
Females demonstrated prolonged ipsilateral mechanical hyperalgesia in the low back region after NGF injections compared with males. When compared to control (PBS), NGF injections (arrows) resulted in ipsilateral low back mechanical hyperalgesia with algometer testing in (**A**) male and (**B**) female rats. (**A**) Male NGF rats had significant decreased thresholds at D2 and D5 + 4 h that persisted through D17 when compared to baseline (D0). (**B**) Female NGF rats had significant decreased thresholds at D2 and D5 + 4 h that persisted through D24 when compared to D0. (**C**) NGF induced a significant mean difference from baseline in ipsilateral low back mechanical thresholds at D2 and D5 + 4 h through D17 in males and D2 and D5 + 4 h through D24 in females. Females in the NGF group had a significant mean difference when compared to the male NGF group at D17, D21, and D24. LBP resolved by D21 in males and D28 in females. Data were analyzed by two−way ANOVA with (**A**,**B**) Bonferroni’s comparisons within groups compared to D0 or (**C**) Tukey’s comparisons between groups for *n* = 8/group. * *p* < 0.01 vs. D0 prior to injection and ^#^ *p* < 0.01 vs. the time−matched vehicle group, ^†^ *p* < 0.01 vs. NGF male ipsilateral.

Male and female control rats had no significant differences in the ipsilateral withdrawal threshold to noxious deep trunk mechanical stimuli compared to D0 at any time point (Figure 4A,B; *p* > 0.05). In contrast, male and female NGF rats demonstrated significant decreases at D2, D5 + 4 h, D7, D10, D14, and D17 in males (Figure 4A; *p* < 0.01) and D2, D5 + 4 h, D7, D10, D14, D17, D21, and D24 in females (Figure 4B; *p* < 0.01). An analysis examining the magnitude of change confirmed that animals receiving NGF, but not control rats, had a significant magnitude of change at D2, D5 + 4 h, D7, D10, D14, and D17 in males and D2, D5 + 4 h, D7, D10, D14, D17, D21, and D24 in females ipsilaterally (Figure 4C). NGF Females also had a magnitude of change for a longer duration (i.e., the mechanical hypersensitivity persisted at D17, D21, and D24 and finally resolved by D28) compared with NGF males (resolution by D21) (Figure 4C).

**Figure 5:**
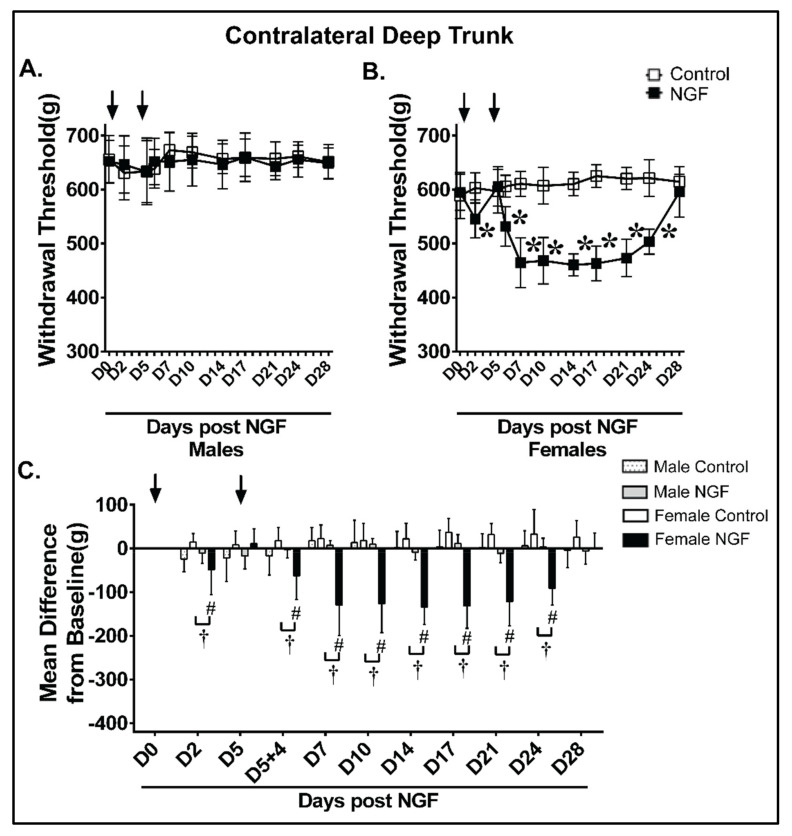
Females demonstrated contralateral low back mechanical hyperalgesia after NGF injections compared with males. When compared to control (PBS), NGF injections (arrows) resulted in contralateral low back mechanical hyperalgesia with algometer testing in (**B**) female but not (**A**) male rats. (**B**) Female NGF rats had significant decreased thresholds by D2 and then D5 + 4 h that persisted through D24 when compared to baseline (D0). (**C**) NGF induced significant changes in the mean difference from baseline thresholds in females when compared to time-matched vehicles and the male NGF group at D2 and then D5 + 4 h that persisted through D24. Contralateral LBP resolved by D28 in females. Data were analyzed by two−way ANOVA with (**A**,**B**) Bonferroni’s comparisons within groups compared to D0 or (**C**) Tukey’s comparisons between groups for *n* = 8/group. * *p* < 0.01 vs. D0 prior to injection and ^#^ *p* < 0.01 vs. time−matched vehicle group, ^†^ *p* < 0.01 vs. NGF male contralateral.

Male and female control rats had no significant alterations to the contralateral withdrawal threshold to deep trunk mechanical hyperalgesia compared to D0 (*p* > 0.05; Figure 5A,B). Male NGF rats had no significant contralateral deep trunk hyperalgesia at any assessed time point (Figure 5A; *p* > 0.05). In contrast and of note, female NGF rats demonstrated a significant decrease at D2, D5 + 4 h, D7, D10, D14, D17, D21, and D24 (Figure 5B; *p* < 0.01). An analysis examining the magnitude of the change confirmed that NGF females, but not control female or NGF male rats, had a significant magnitude of change at D2, D5 + 4 h, D7, D10, D14, D17, D21, and D24 contralaterally (Figure 5C). The contralateral deep trunk mechanical hyperalgesia resolved in females by D28 (Figure 5B,C).

## 5. Discussion

This study characterized and compared local (trunk) and remote (hindpaw) cutaneous and deep mechanical sensitivity/hyperalgesia in a unilateral NBF-LBP model in adult male and female rats. To our knowledge, this is the first study to examine potential sex differences in the unilateral NGF-LBP rat model, which is translationally important, as females clinically report greater LBP frequency, intensity, and anatomical distribution than males [42,43]. Our findings indicated that female NGF rats had a substantial reduction in the cutaneous and deep trunk mechanical withdrawal threshold bilaterally, while males exhibited only localized (ipsilateral) changes in trunk mechanical hypersensitivity. Moreover, the resolution of trunk mechanical hypersensitivity required an additional 7 days in female NGF rats compared to NGF males. It should be also noted that there was no remote (hindpaw) cutaneous mechanical hypersensitivity found in either female or male NGF rats.

The present study yielded several important behavioral findings. First, we found that there were similar cutaneous and deep trunk mechanical behavioral sensitivity baselines between males and females for all behavioral assays performed. Second, females demonstrated a persistent ipsilateral cutaneous trunk mechanical hypersensitivity, as demonstrated by VF testing (1 g and 4 g), which lasted 3 days longer (1 g) and 7 days longer (4 g) than males, respectively. Contralateral cutaneous mechanical hypersensitivity was demonstrated in NGF females at D10 and D14 (1 g) and D7-D17 (4 g). NGF females differed from NGF males because males did not demonstrate hypersensitivity to a contralateral cutaneous low back mechanical stimulus at any time point. In all cases (i.e., both NGF males and NGF females), the onset of cutaneous mechanical sensitivity (D7 and D10) was delayed compared to the onset of deep trunk mechanical hyperalgesia (D5 + 4 h) following the second NGF injection. In contrast, the resolution of sensitivity to a 4 g cutaneous mechanical stimulus typically followed the resolution of deep trunk mechanical hyperalgesia in males (D21) and females (D28), respectively. Collectively, these cutaneous hypersensitivity findings are consistent with processes such as secondary hyperalgesia or allodynia, which typically manifest in patients with LBP [35,36]. Those changes are hypothesized to occur, in part, as a result of pathophysiological changes in the spinal cord consistent with central sensitization [35,36].

Similar to the cutaneous trunk mechanical stimulation findings, NGF male rats did not demonstrate contralateral decreases in the withdrawal threshold to deep trunk algometer testing, supporting previously reported findings [28]. However, and importantly, contralateral deep trunk mechanical sensitivity reductions in female NGF rats occurred at D2, D5 + 4 h, D7, D10, and D14, which parallels the hyperalgesia findings on the ipsilateral side. When the magnitude of change on the ipsilateral side was compared to the contralateral side in female NGF rats, significant differences were noted at D2 and D5 + 4 h. This suggests that contralateral deep mechanical hypersensitivity is less pronounced during the earlier phases of this NGF-LBP model and peaks later in the time course than the ipsilateral side.

Third, there was no detected cutaneous mechanical hypersensitivity in the hindpaws of any tested group. This contrasts to an earlier unilateral NGF-LBP study using females in which NGF females exhibited ipsilateral but not contralateral cutaneous hindpaw sensitivity on D12 compared to D0 [32]. Possible differences in testing methodology, test environment, and/or the noted level of variability in this measure of remote cutaneous hypersensitivity were most likely responsible for the discrepancy between these two NGF-LBP studies using female animals. It should also be noted that modifications of the original NGF-LBP methodology [28], such as using bilateral NGF injections, different NGF concentrations, and/or different species (mice, rabbits, and felines) may likely result in more widespread and/or remote/distant mechanical hypersensitivities over different timelines [44].

In this NGF-LBP model, we examined several potential variables that revealed differences between males and females, including the time to LBP resolution, laterality, and/or the magnitude/severity of change, all of which are hypothesized to contribute to females being more sensitive to musculoskeletal pain states. Females are thought to have different pain experiences from males, and one way this might occur is through prolonged pain states or a slower resolution of pain. Here we examined the resolution of LBP in males and females by extending the previously reported experimental time course to detect the resolution of LBP behaviorally. In human LBP patients, one consistent somatosensory alteration associated with LBP is a hypersensitivity to a noxious algometer mechanical stimulus to the low back. In the current study, the small animal algometer test on the side of NGF injections (ipsilateral paraspinal region at the L5 level) was used, and it was found that NGF males had mechanical hyperalgesia that continued through D17 with the resolution of LBP by D21. In contrast, females demonstrated ipsilateral low back mechanical hyperalgesia that continued beyond D24 and resolved by D28. These data extend previous findings using the same model and are the first to demonstrate that, compared to NGF males, NGF females have prolonged ipsilateral deep trunk mechanical hyperalgesia. This is an important foundational finding in the NGF-LBP model, and future studies should seek to identify the underlying mechanisms responsible for these sex-related differences.

The current findings agree with the results of other studies where different preclinical models of pain were used, suggesting females can have different mechanistic and behavioral manifestations than males. In preclinical pain models, there is evidence that some of the biological underpinnings leading to a more prolonged pain state could be related to the immune responses to pain, both in the periphery, where the injury/pain mediator interacts with the primary afferent, and in the spinal cord. For example, in a mouse model of complete Freund’s adjuvant injected into the whisker pad, there were reported sex differences in CGRP, BDNF, and proinflammatory cytokine expression between male and female mice in the trigeminal ganglia, representing a mechanism by which peripheral immune responses between males and females differ [45]. Males and females have also been shown to differ with respect to the neurokinin 1 internalization of nociceptors, a marker of peripheral pain signaling and another route by which peripheral pain modulation may differ between the sexes [46]. In the spinal cord, a neuropathic murine model found that males recovered faster (81 days) compared to females (unresolved after 121 days) and that the extended recovery time of females was associated with longstanding microglial and astrocytic hyperactivation (neuroimmune activation) within the spinal cord [47]. Potential glial-mediated mechanisms could explain, in part, how females undergo prolonged LBP in response to NGF compared with males.

Collectively, cutaneous and deep trunk mechanical hypersensitivity in the unilateral NGF-LBP model is different in males and females. Females, in general, are more sensitive to this NGF-induced muscle pain, with prolonged, bilateral, and sometimes more severe LBP, as purported by the mechanical sensitivity behavior assays. Greater mechanical sensitization was recently reported in women compared to men following NGF injections into the human masseter muscle [8]. This clinical study supports the sex-related differences found in the current NGF-induced LBP study. The underlying biological mechanisms for this are unknown. However, there are several potential explanations (for a greater, more detailed review see [27,48,49,50]). We hypothesize that the sex differences in rat NGF-induced mechanical hypersensitivity could be due to one or more of the following: (1) Differences in central sensitization. A larger affected area of the spinal cord due to a greater activation of adaptive immune cells and infiltration in females versus the activation of the innate immune cells in males may account for the behavioral differences we observed. The recent discovery of a sexual dimorphism in male and female mice and how they achieve mechanical hypersensitivity via either the innate (female) or adaptive (male) immune response in the spinal cord [51] provides a compelling mechanism to explain the contralateral mechanical hypersensitivity in females. In humans, NGF has been found to be produced and released from CD14+ T-cell clones and monocytes [52,53]. The time delay and magnitude of the contralateral sensitivity also aligns well with the process being dependent on an immune-related and/or diffusion process. In male rats, the early stages of pain development, wherein primary afferent priming via NGF exposure leads to spinal neuronal sensitization in this unilateral NGF-LBP model, depend initially on microglial activation and are maintained by astrocyte activation [29,54]. Additional work on the presence, level, and mechanisms of immune cell activation throughout the spinal cord in male and female rodents should be considered [7]. (2) Differences in peripheral sensitization. It was previously found that estrogen receptors colocalize with NGF receptors (i.e., trkA and p75) in laminae I and II of the spinal cord and the dorsal root ganglion [55,56]. Estrogen was also found to differentially regulate both the p75 and trkA NGF receptors, with estrogen upregulating trkA and bi-directionally modulating p75 based on the environment [55,56]. It is plausible that estrogen is able to affect the ability of NGF to produce prolonged mechanical hypersensitivity through the increased expression of NGF, NMDA, and/or NR2B receptors [8,27,49,57]. Estrogen is known to mediate increases in the peripheral expression of NMDA receptors [57], and NGF injection into the human masseter muscle induced an increased expression of NMDA receptors by the masticatory muscle nerves in women [8]. Additionally, differences in the peripheral immune response to noxious stimuli, such as the levels of CGRP, BDNF, and proinflammatory cytokines, may account for differences in the peripheral sensitization of nociceptors between the sexes [45,58]. (3) Differences in the descending modulation of pain. Pain responses from the brain, such as the descending facilitation of spinal nociceptive processing and signaling, are oftentimes implicated in the maintenance of persistent pain by promoting spinal/central sensitization via descending pathways from the brainstem [10]. In the rat, an intramuscular injection of hypertonic saline to the gastrocnemius muscle has previously been found to result in bilateral pain responses in females but not in males [59]. This bilaterality was not fully explained by studies in the periphery and spinal cord [59]. The role of the descending modulation of pain was examined via lesions of the noradrenergic and serotonergic cells of the supraspinal descending modulation system and it was found to be differently involved in both sexes [59]. The authors attributed the bilaterality of female pain behavior to the ability of females to elicit stronger descending facilitation of nociception in the spinal cord (trigger the threshold of descending facilitation) following exposure to a painful mediator in the muscle (hypertonic saline) than males [59]. Furthermore, the organization of the periaqueductal gray and descending pain modulatory pathways is known to be sexually dimorphic, contributing to the differences in opioid medication effects in males and females [10,60]. (4) Differences in the collateral sprouting and/or nociceptor innervation of tissue. Although NGF is typically more associated with nociceptive sprouting and controlling innervation density during embryonic development, there is evidence that increased concentrations of NGF can elicit the sprouting of axon terminals and increase the innervation density of target tissues, especially during pathological states [61,62,63,64]. However, intradermal and intramuscular NGF injections failed to increase muscle nerve fiber density in males and females [8,33]. Investigations of these hypotheses would be important first steps to begin to uncover the underlying mechanisms resulting in the behavioral sex differences in the NGF model of LBP.

## 6. Conclusions

The current study using the NGF-LBP model revealed behavioral sex differences with prolonged, bilateral, and sometimes more severe somatosensory alterations in females compared to males. These findings provide substantial support for future mechanistic studies to determine the neurobiological underpinnings for this difference. Such studies may help reveal additional meaningful therapeutic targets to impact the global problem of LBP.

## Data Availability

The data presented in this study are available on reasonable request from the corresponding author. The data are not publicly available due to security reasons.

## Supplementary Materials

The following supporting information can be downloaded at: https://www.mdpi.com/article/10.3390/biology11060924/s1, Figure S1: Females had a similar severity of ipsilateral and a greater severity of contralateral mechanical hypersensitivity to a 1 g cutaneous stimulus during persistent low back pain compared with males. (A) When compared to control (PBS), NGF injections (arrows, 0.8 μM) resulted in ipsilateral low back mechanical hypersensitivity with an increased mean difference from baseline to a cutaneous 1 g filament stimulus from D7 to D14 in male rats and from D7 to D17 in female rats. No significant differences were found between males and females at any time points. (B) NGF injections (arrows, 0.8 μM) resulted in contralateral low back mechanical hypersensitivity with an increased mean difference from baseline to a cutaneous 1 g filament stimulus at D10 and D14 in female rats. Females had significantly greater responses compared with males at D10 and D14. Data are reported as means ± SD and analyzed by two-way ANOVA with Tukey’s multiple comparisons between groups for *n* = 8/group. * *p* < 0.01 vs. vehicle at the same time point and # *p* < 0.01 vs. NGF male contralateral at the same time point; Figure S2: Females demonstrated a similar severity of ipsilateral and a greater severity of contralateral mechanical hypersensitivity to a 4 g cutaneous low back stimulus during persistent low back pain compared with males. (A) When compared to control (PBS), NGF injections (arrows, 0.8 μM) resulted in ipsilateral low back mechanical hypersensitivity with an increased mean difference from baseline to a cutaneous 4 g filament stimulus from D7 to D17 in male rats and from D7 to D24 in female rats. No significant differences were found between males and females at any time points. (B) NGF injections (arrows, 0.8 μM) resulted in contralateral low back mechanical hypersensitivity with an increased mean difference from baseline to a cutaneous 4 g filament stimulus at D7 through D17 in female rats. Females had significantly greater responses compared with males at D10, D14, and D17. Data are reported as means ± SD and analyzed by two-way ANOVA with Tukey’s multiple comparisons between groups for *n* = 8/group. * *p* < 0.01 vs. vehicle at the same time point and # *p* < 0.01 vs. NGF male contralateral at the same time point.
