# Supplementary material for: Sex-Related Pain Behavioral Differences following Unilateral NGF Injections in a Rat Model of Low Back Pain
Source: Biology (Basel). 2022 Jun 16;11(6):924. doi: 10.3390/biology11060924 (PMC9219698; doi:10.3390/biology11060924)
Supplement: Supplementary file 1 [file biology-11-00924-s001.zip › Biology Suppl Figures/biology-1673655 - Original.pdf]

Supplementary Materials:

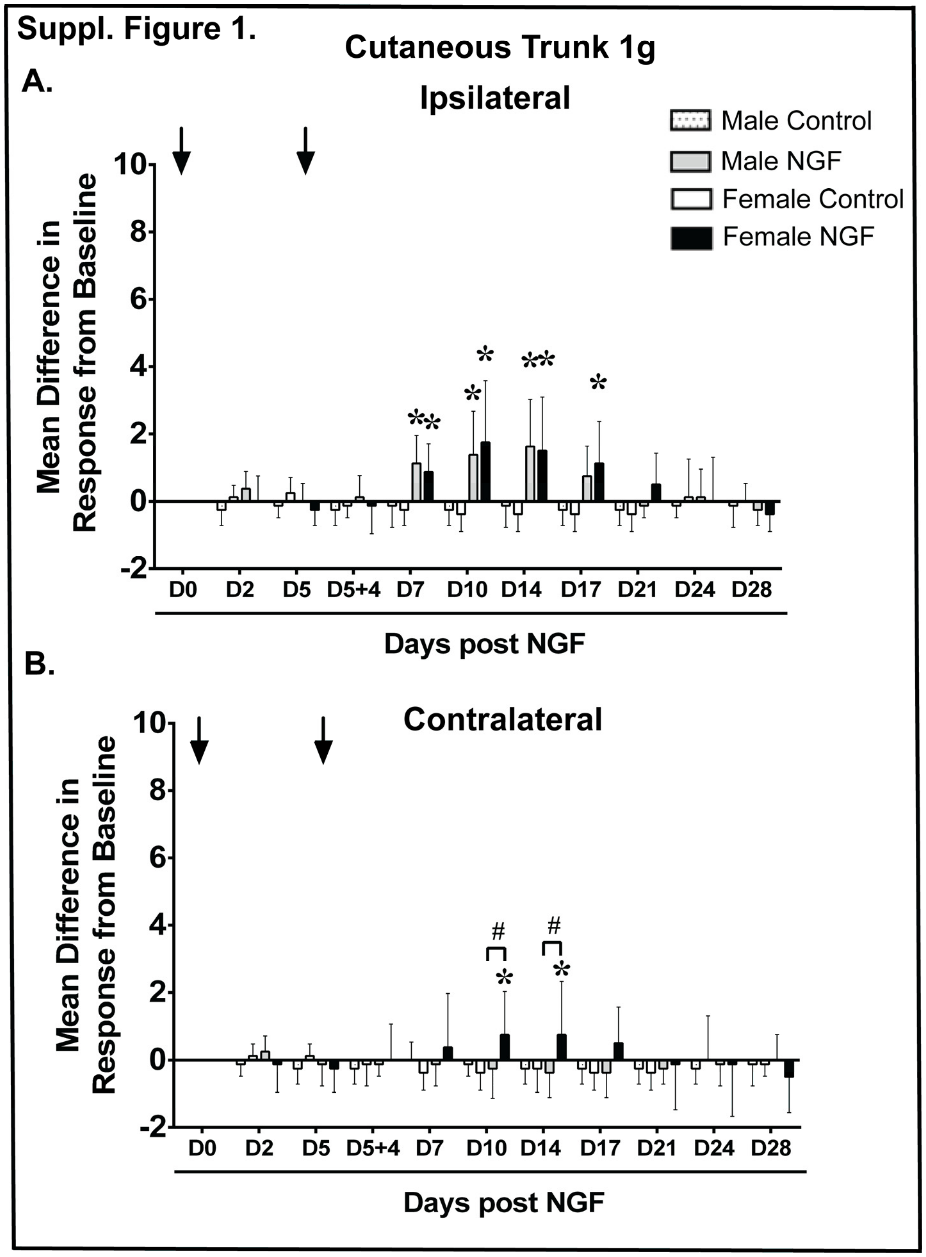

Suppl. Figure S1. Females had similar severity of ipsilateral and greater severity of contralateral mechanical hypersensitivity to a 1g cutaneous stimulus during persistent low back pain compared with males. A) When compared to control (PBS), NGF injections (arrows, 0.8 µM) resulted in ipsilateral low back mechanical hypersensitivity with an increased mean difference from baseline to a cutaneous 1g filament stimulus from D7 through D14 in male rats and D7 through D17 in female rats. No significant differences were found between males and females at any time points. **B)** NGF injections (arrows, 0.8 µM) resulted in contralateral low back mechanical hypersensitivity with an increased mean difference from baseline to a cutaneous 1g filament stimulus at D10 and D14 in female rats. Females had significantly greater responses compared with males at D10 and D14. Data are reported as mean ± SD and analyzed by two-way ANOVA with Tukey's multiple comparisons between groups for n=8/group. *P<0.01 vs. vehicle at the same time point and $^{\#}P$<0.01 vs. NGF male contralateral at the same time point.

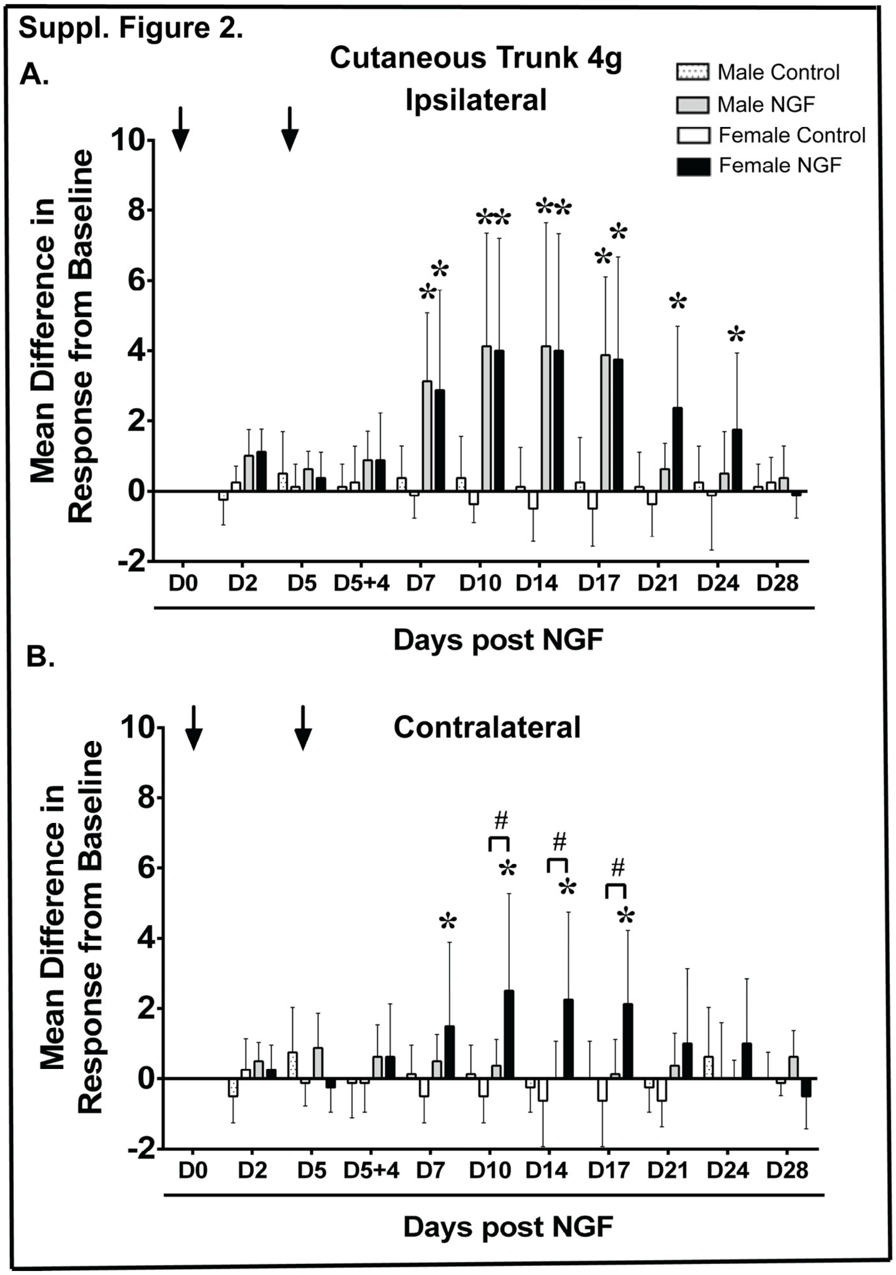

Suppl. Figure S2. Females demonstrated similar severity of ipsilateral and greater severity of contralateral mechanical hypersensitivity to a 4g cutaneous low back stimulus during persistent low back pain compared with males. A) When compared to control (PBS), NGF injections (arrows, 0.8 μM) resulted in ipsilateral low back mechanical hypersensitivity with an increased mean difference from baseline to a cutaneous 4g filament stimulus from D7 through D17 in male rats and D7 through D24 in female rats. No significant differences were found between males and females at any time points. **B)** NGF injections (arrows, 0.8 μM) resulted in contralateral low back mechanical hypersensitivity with an increased mean difference from baseline to a cutaneous 4g filament stimulus at D7 through D17 in female rats. Females had significantly greater responses compared with males at D10, D14, and D17. Data are reported as mean ± SD and analyzed by two-way ANOVA with Tukey's multiple comparisons between groups for n=8/group. *P<0.01 vs. vehicle at the same time point and $^{\#}P$<0.01 vs. NGF male contralateral at the same time point.
