# Supplementary figures and images for: Sex-Related Pain Behavioral Differences following Unilateral NGF Injections in a Rat Model of Low Back Pain
Source: Biology (Basel). 2022 Jun 16;11(6):924. doi: 10.3390/biology11060924 (PMC9219698; doi:10.3390/biology11060924)

Suppl. Figure 1.

Cutaneous Trunk 1g

A.

Ipsilateral

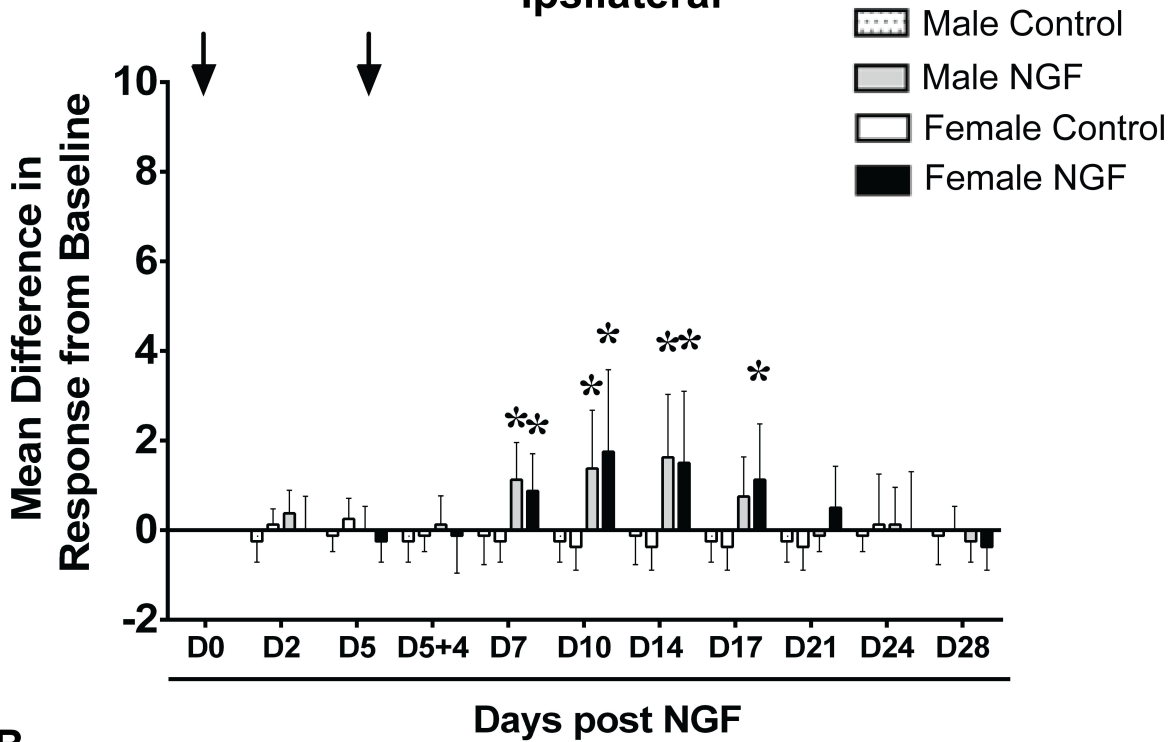

B.

Contralateral

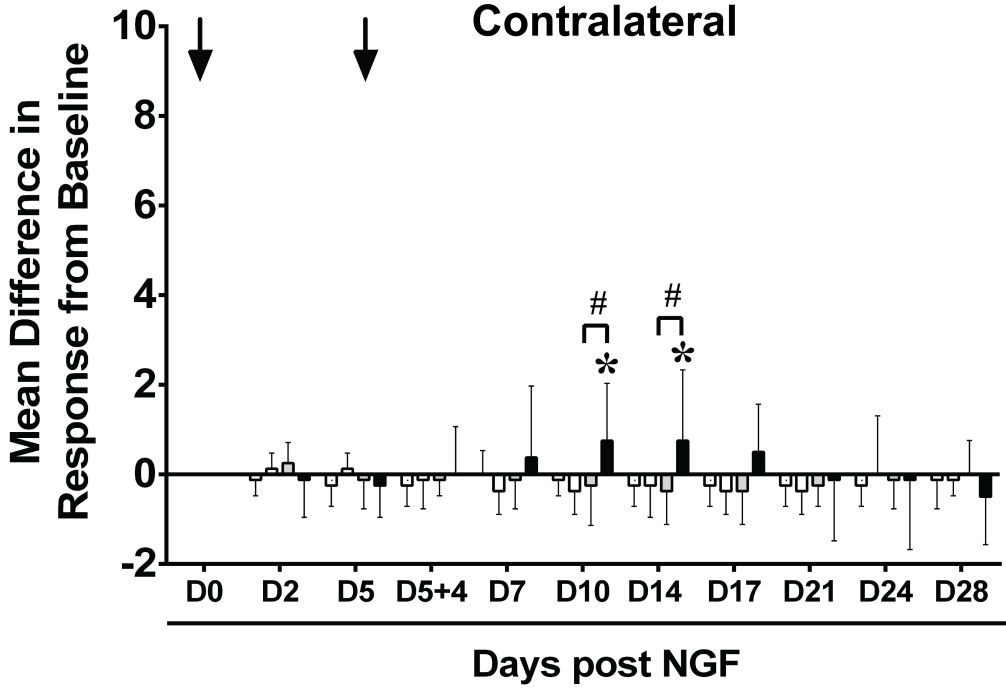

Supplement: Supplementary file 1 [file biology-11-00924-s001.zip › Biology Suppl Figures/Suppl. Figure S1.pdf]

Suppl. Figure 2.

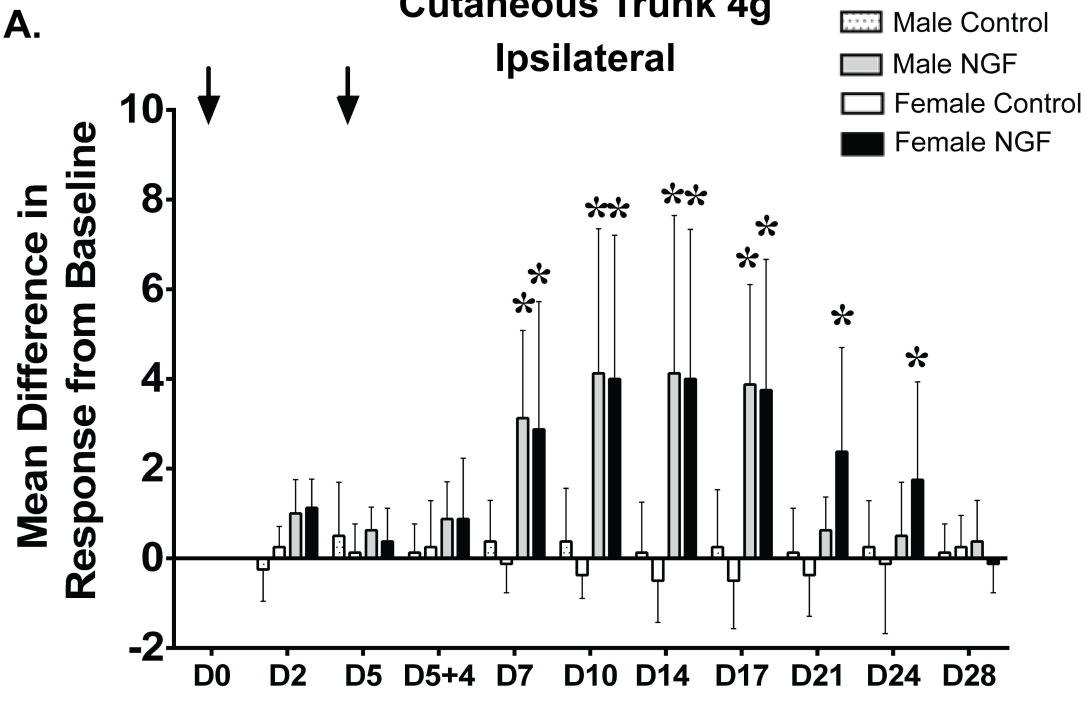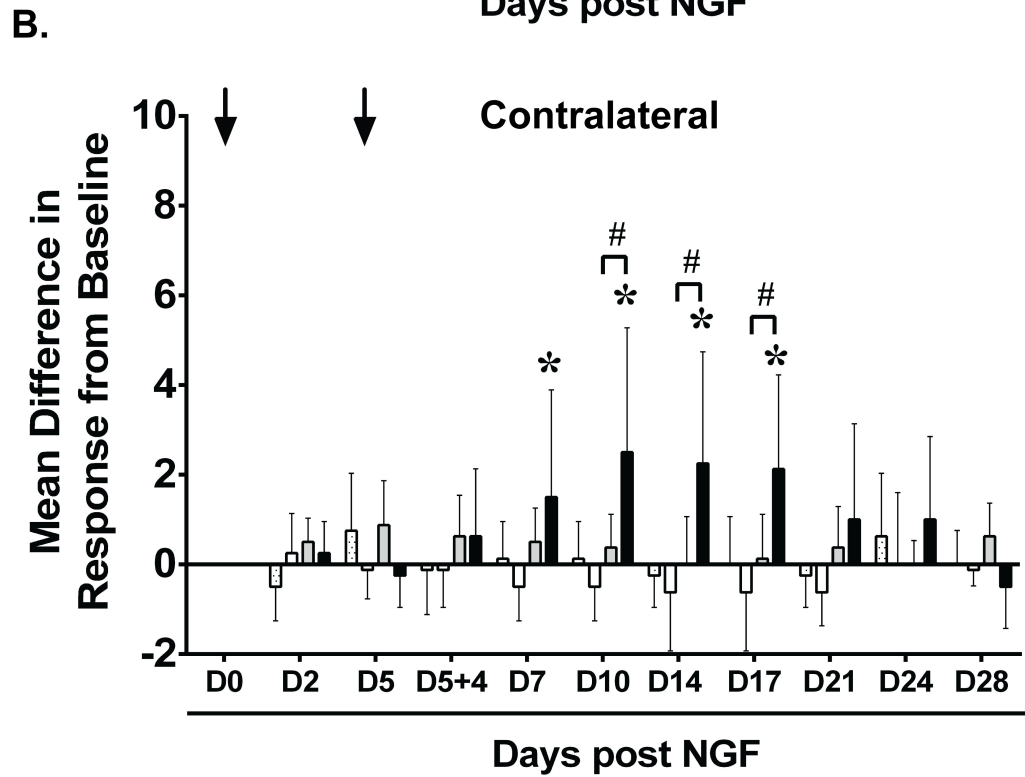

Supplement: Supplementary file 1 [file biology-11-00924-s001.zip › Biology Suppl Figures/Suppl. Figure S2.pdf]
